# Current Antibiotic Resistance Trends of Uropathogens in Central Europe: Survey from a Tertiary Hospital Urology Department 2011–2019

**DOI:** 10.3390/antibiotics9090630

**Published:** 2020-09-22

**Authors:** Jan Hrbacek, Pavel Cermak, Roman Zachoval

**Affiliations:** 1 Department of Urology, 3rd Faculty of Medicine, Charles University and Thomayer Hospital, Videnska 800, 140 59 Prague, Czech Republic; roman.zachoval@ftn.cz; 2 Department of Clinical Microbiology, Thomayer Hospital, Videnska 800, 140 59 Prague, Czech Republic; pavel.cermak@ftn.cz

**Keywords:** antibiotics, Enterobateriaceae, resistance, urinary tract infection, *E. coli*, *Klebsiella*, *Proteus*, *Pseudomonas*, *Enterococcus*

## Abstract

Monitoring of pathogen resistance profiles is necessary to guide empirical antibiotic therapy before culture and sensitivity results become available. The aim of this study was to describe current antibiotic resistance patterns of five most frequent causative uropathogens in a Department of Urology of a tertiary referral centre in Central Europe over a period of nine years. The Hospital Department of Clinical Microbiology database was used to extract data on all positive urine samples from inpatients in the Department of Urology between 2011 and 2019. Numbers of susceptible and resistant isolates per year were calculated for five most frequent uropathogens: *Escherichia coli, Enterococcus* spp., *Klebsiella* spp., *Pseudomonas aeruginosa*, and *Proteus* spp. Antimicrobial agents selected for the survey included: ampicillin, amoxicillin/clavulanic acid, piperacillin/tazobactam; cefuroxime, cefotaxime, ceftazidime and cefepime; ciprofloxacin and ofloxacin; gentamicin and amikacin; ertapenem, meropenem and imipenem; trimethoprim-sulfamethoxazole (co-trimoxazole), nitrofurantoin, colistin, and vancomycin. High resistance rates of Gram-negative uropathogens were demonstrated to most common antimicrobials, with statistically significant increasing or decreasing trends in some cases. No carbapenem-resistant *Enterobacteriaceae* were isolated. Vancomycin-resistant *Enterococcus* spp. strains were rare in our population.

## 1. Introduction

Less than a century after Sir Alexander Flemming’s discovery of *Penicillinum notatum*, the growing antimicrobial resistance (AMR) of most clinically relevant bacteria to antimicrobial agents represents a major challenge to the public health. AMR is caused by a mixture of factors, from improper antibiotics use in health care, to their unrestrained use in agriculture [1]. AMR has become a widespread phenomenon (albeit with important spatial and temporal differences) occurring in all parts of the world, including Europe [2,3,4], the Americas [5,6,7], Africa [8], and Australasia [9,10,11]. In the United States, more than 2.8 million people acquire an antibiotic-resistant infection every year, resulting in more than 35,000 deaths; the corresponding figure for Europe is 33,000 annually [12]. Besides the associated morbidity and mortality, infections by multi-drug-resistant pathogens increase the pressure on heath care systems, too: An episode of a carbapenem-resistant Enterobacteriaceae urinary tract infection has been estimated to cost the health care system 66,000 USD [13].

Urinary tract infections (UTIs) belong among the most common types of infectious disease, accounting for approximately 150–250 million cases globally per year [14]. They are usually caused by the host’s endogenous microbial flora and Gram-negative enteric rods, such as *E. coli, Klebsiella, Proteus* etc., are their most frequent etiological agents [15]. Approximately 50% of women acquire a UTI at least once in their lifetime and recurrences are common [15]. UTIs are therefore an economic problem, too: The costs of treatment of adult female UTIs were estimated at 58 million euro in France alone in 2012 [16]. A complicated UTI (one associated with structural or functional abnormalities of the urinary tract, immunocompromised host, virulent microorganism, etc.) [15] may cost the health care system between 4028 and 7740 euro [17].

Antibiotic consumption is a primary driver for AMR, a fact documented on a hospital, regional, and country level [18,19]. A north-to-south gradient in AMR exists in Europe [20], with southern countries having higher rates of antibiotic-resistant UTIs. Perhaps not coincidentally, all four top antibiotic consumers (Greece, Cyprus, France, and Italy) belong among southern European states [21].

Despite the existence of international guidelines, research shows improper antibiotic prescribing is commonplace [22]. According to a survey from the United States, 30% of primary care antibiotic prescriptions were classified as inadequate [23]. Chardavoyne et al. reported appropriate antibiotic treatment in 68% of adult cystitis cases and 46% of pyelonephritis cases [24]. Treatment in the absence of infection is common [24] and although not recommended in international guidelines for lower UTI management, fluoroquinolones are frequently prescribed [25].

In clinical practice of the past, the problem of AMR used to be overcome by the advent of new more potent antimicrobials. This is no longer the case. Many pharmaceutical companies have withdrawn from the field, leaving the antibiotic pipeline virtually empty. There are multiple reasons for this that are out of the scope of this paper (high cost of antibiotic development, availability of inexpensive generic antimicrobials, antibiotic stewardship reducing overall antibiotic use, and regulated use of new antibiotics to maintain their efficiency) [26]. The few antimicrobials under development are mostly derivatives of the major existing antibiotic classes (particularly beta-lactams, beta-lactamase inhibitors, and tetracyclines) and all exhibit some degree of pre-existing cross-resistance [27]. Antimicrobial agents with non-traditional therapeutic targets (virulence factors inactivators, inhibitors of adhesion and biofilm formation, monoclonal antibodies against bacterial exotoxins, quorum-sensing and other communication channels disrupting agents or those countering immune evasion) are being developed but not yet ready for clinical use [28]. The same holds true for alternative substances with antimicrobial effect, such as zinc-oxide nano-particles [29], cell-free probiotic suspensions [30], essential oils [31], or phage therapy [32].

As stated above, flaws in antibiotic prescribing are among the causes of increasing AMR. The knowledge of local and regional antimicrobial susceptibility patterns is one of the ways to improve antibiotic prescription and at least in part to counter the development of AMR. The aim of this study was to describe the antibiotic resistance patterns of the five most frequent causative uropathogens in a Department of Urology of a tertiary referral centre in Central Europe over a period of nine years.

## 2. Results

**Figure 1:**
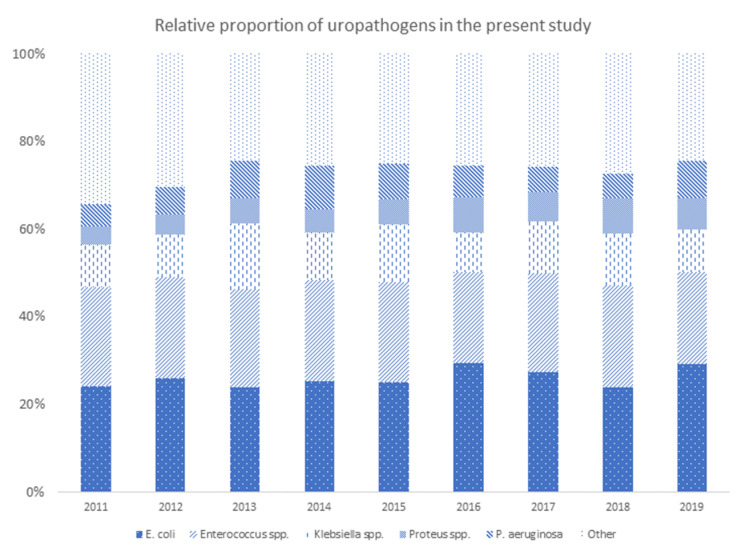
Spectrum of uropathogens during the study period (relative proportions).

From a total of 25,593 urine cultures from inpatients reported between 1 January 2011 and 31 December 2019, 6897 (26.9%) samples yielded a positive result. Spontaneous mid-stream urine samples represented 49.3% of all positive urine cultures; 33.8% were catheterised urine specimens; 8.9% originated from nephrostomy tubes; and suprapubic catheter, ureteroileostomy, ureteric catheter, and “not specified” represented 1.3%, 4.6%, 1.6%, and 0.4% of all positive urine samples, respectively. *E. coli* was the most frequent bacteria isolated (26.0%), followed by *Enterococcus* spp. (22.4%), *Klebsiella* spp. (11.3%), *P. aeruginosa* (7.3%), and *Proteus spp.* (6.2%). The relative prevalence of these microorganisms changed somewhat over time, with *E. coli* and *Proteus* spp. showing an increase in prevalence (*p* = 0.049 and *p* < 0.0001, respectively) (Figure 1). Table 1 outlines the cumulative resistance rates for individual uropathogens and antibiotics, respectively, for the entire study period.

### 2.1. Penicillin Derivatives

*E. coli* and *Proteus* spp. resistance rates for ampicillin exceeded 50%, precluding its empirical use for the treatment of UTIs. *Enterococcus* spp. resistance to ampicillin more than doubled, from 7.2% to 16.3% (*p* for trend 0.013). Resistance to amoxicillin/clavulanic acid was above 10% for *E. coli* and *Proteus* spp. in most study years and ranged between 20.4% and 58.9% for *Klebsiella* spp. with a significant increasing trend (*p* = 0.041). Resistance rates for all Gram-negatives in the study decreased significantly for piperacillin/tazobactam during the study period (Supplementary Material Table S1). Supplementary Material Figure S1a–e show statistically significant trends in antimicrobial resistance for each of the five uropathogens covered in this survey.

### 2.2. Cephalosporines

Among cephalosporines, cefuroxime resistance rates fluctuated above 10% and 15% for *E. coli* and *Proteus* spp., respectively. *Proteus* spp. showed a significant decrease in resistance to cefuroxime (*p* = 0.012). Cefotaxime and ceftazidime had a slightly more favourable resistance profile for *E. coli* and *Proteus* spp. (<10% in most of the study period) than cefuroxime. Over 30% of the *Klebsiella* spp. strains tested were resistant to cefuroxime, cefotaxime, and ceftazidime. Cefepime resistance rates were above 25%, 50%, and 30% for *E. coli*, *Klebsiella* spp., and *P. aeruginosa*, respectively, in most study years despite a significant decreasing trend (Supplementary Material Table S2).

### 2.3. Fluoroquinolones

Ciprofloxacin susceptibility was tested on Gram-negatives only; resistance rates fluctuated above 40% but showed a consistently decreasing trends for all of them except *Proteus* spp. (Supplementary Material Table S3). Resistance to ofloxacin exceeded 25% in most examined years for *E. coli*, 35% for *Klebsiella* spp., and 40% for *Proteus* spp., with an increasing trend for the latter two.

### 2.4. Aminoglycosides

The *E. coli* resistance rate to gentamicin did not exceed 10% (with one year’s exception) but was above 30% for all other Gram-negatives surveyed. Amikacin showed a better resistance profile with >95% susceptible isolates of *E. coli*, *Klebsiella* spp., and *Proteus* spp.

The *P. aeruginosa* resistance rate against amikacin was 9% compared to 31% for gentamicin. Both exhibited decreasing trends in resistance (*p* = 0.046 and *p* = 0.006 for gentamicin and amikacin, respectively; see Supplementary Material Table S4 for details).

### 2.5. Carbapenems

*E. coli*, *Klebsiella* spp., and *Proteus* spp. isolates were all susceptible to ertapenem, meropenem, and imipenem except one *E. coli* and nine *Klebsiella* spp. strains resistant to ertapenem during the entire study period. *P. aeruginosa* showed a >20% resistance rate for both meropenem and imipenem in most study years despite a decreasing trend in the case of meropenem (*p* = 0.002) (Supplementary Material Table S5).

### 2.6. Co-Trimoxazole and Nitrofurantoin

Co-trimoxazole resistance for all Gram-negatives far exceeded 30% without any demonstrable trend. *E. coli* and the only Gram-positive pathogen in the survey, *Enterococcus* spp., were resistant to nitrofurantoin in less than 10%, and often less than 5% of strains. *Klebsiella* spp. resistance exceeded 50% and was on the increase up to 83% in 2019 (*p* for trend 0.002) (Supplementary Material Table S6).

### 2.7. Vancomycin and Colistin

*Enterococcus* resistance to vancomycin remained low despite an increasing trend (*p* = 0.018). All *P. aeruginosa* isolates were susceptible to colistin. There were three individual isolates of *E. coli* and *Klebsiella* spp. displaying colistin resistance (Supplementary Material Table S7).

## 3. Discussion

In the present study, we report the AMR rates for five major causative agents of UTIs. These data, together with other papers in the literature, complement the picture of AMR worldwide and its trends. The resistance rates of Gram-negative uropathogens to a majority of commonly used antimicrobials in the present study were high, a fact to be expected given the epidemiological situation in the world today [2,3,4,5,6,7,8,9,10,11]. With increasing individual mobility and international travel easier than ever, a global spread of multi-drug-resistant bacterial strains seems an inevitable reality [13].

*E. coli* resistance to most antimicrobials surveyed in our study approached or exceeded 30% in the case of ampicillin, both fluoroquinolones, cotrimoxazole, and was above 10% for amoxicillin/clavulanate, piperacillin/tazobactam, cefuroxime, and cefepime. Third-generation cephalosporines proved reasonably efficient as well as gentamicin and nitrofurantoin. Resistance rates approached 0% for amikacin, carbapenems, and colistin. In a Hungarian study by Magyar et al., similar ranges of resistance for penicillins, cefuroxime, cefotaxim, aminoglycosides, carbapenems, co-trimoxazole, and nitrofurantoin were reported [33]. Our isolates were, however, twice as resistant to ciprofloxacin and five times more resistant to cefepime. Our data show twice higher resistance rates for ciprofloxacin and cefuroxime than an older survey (1999–2009) from Ireland [4].

During the study period, 8.7% of *E. coli* strains were reported to be producers of extended-spectrum beta-lactamase (ESBL; yearly incidence 5.4% to 12.3%, no significant trend observed). This compares to 5.4% and 10.5% of ESBL producers in a recent British and Moroccan study, respectively [34,35]. Some authors report excellent rates of susceptibility to nitrofurantoin and fosfomycin among ESBL-producing *E. coli* [2,34,35,36,37]; these antimicrobials should be used for the treatment of uncomplicated UTIs only. Most inpatients will, however, be classified as having a complicated UTI, and their therapeutic options will be limited to reserve antibiotics, such as carbapenems or amikacin [34].

Alarming rates of 30% to 90% resistance were demonstrated for *Klebsiella* spp. against all penicillin derivatives, cephalosporins, fluoroquinolones, co-trimoxazole, nitrofurantoin, and gentamicin. Amikacin and carbapenems seem the only suitable antimicrobials that should be considered for empirical treatment of complicated UTIs caused by *Klebsiella* spp. in our patient population. Resistance rates reported in the Hungarian study are essentially similar [33]. Fajfr et al. reported comparable resistance rates for cefuroxime and penicillin/beta-lactamase inhibitor combinations as we do and “only” 47% of *Klebsiella* strains were resistant to ciprofloxacin [37]. This may be because their study included outpatients whose bacterial isolates are presumed to be generally more susceptible.

*Klebsiella* spp. is the genus with the highest proportion of ESBL-producing isolates [35]. In fact, 33.9% of *Klebsiella* spp. strains in our study were ESBL producers (between 15.4% and 52.3% in the study period; no significant trend demonstrated). This compares to 18.6% of ESBL-producing *Klebsiella* isolates from inpatients in a British report [34] and 25.8% within a mixed population of in- and outpatients in a Moroccan study [35]. Intriguingly, a study of urine cultures from 14 urology services in the Netherlands covering the decade 1998–2009 reported a much more favourable *Klebsiella* spp. antimicrobial susceptibility pattern with resistance rates one order of magnitude lower (piperacillin/tazobactam, cefepime, cefotaxime, ciprofloxacin, gentamicin) than our and other authors’ data [38].

*Proteus* spp. is a typical nosocomial pathogen that has been isolated particularly from patients with complicated UTIs. *Proteae* (a tribe including *Proteus*, *Morganella* spp., and *Providentia* spp.) are intrinsically resistant to nitrofurantoin, colistin, and have decreased susceptibility to imipenem [39]. In our patient population, *Proteus* spp. retained susceptibility to piperacillin/tazobactam, third- and fourth-generation cephalosporins, amikacin, and carbapenems. Resistance rates for all other antibiotics make them unsuitable for empirical treatment of *Proteus*-caused UTIs.

*P. aeruginosa* belongs to the so-called ESKAPE pathogens (*Enterococcus faecium, Staphylococcus aureus, K. pneumoniae, Acinetobacter baumannii, P. aeruginosa*, and *Enterobacter* spp.) [11] that have been associated with serious health care-associated infections worldwide and frequently display a multi-drug-resistant phenotype. It is intrinsically resistant to many antibiotics due to complementary mechanisms, including low outer membrane permeability, AmpC-beta-lactamase production, and the production of several efflux systems. In addition, it can acquire other resistance determinants, such as beta-lactamases and carbapenemases [40], and its survival is enhanced by biofilm formation [41]. In the present study, *P. aeruginosa* displayed resistance rates exceeding 30% for most antimicrobials, including heavy-weight agents, such as piperacillin/tazobactam, cefepime, or meropenem. No isolate was resistant to colistin and a relatively reasonable resistance fluctuating around 10% was demonstrated to amikacin. Magyar et al. reported *P. aeruginosa* resistance rates roughly 50% lower for most antibiotics where a comparison was possible; good susceptibility to amikacin and colistin was also noted. Furthermore, their group reported a statistically significant decrease in *P. aeruginosa* resistance to ciprofloxacin (from 38% to 13%, *p* = 0.025), a trend corroborated by our data (*p* = 0.009).

Against our own expectations, the data show a statistically significant decrease in resistance to ciprofloxacin for *E. coli*, *Klebsiella* spp., and *P. aeruginosa* in the nine years covered by our study. Nonetheless, their resistance rates remain too high to justify their use for empirical treatment of UTIs. An explanation of this unexpected finding may be a reasonable antibiotic prescribing policy in the department: Intravenous fluoroquinolones are seldom prescribed (never as first-line treatment) and they have not been used for preoperative prophylaxis in the department in the past 15 years. Of note, a warning has recently been issued by the European Medicines Agency discouraging the use of fluoroquinolones [42], and although not strictly forbidden, their administration and prescription should happen on a discernible benefit-to-harm ratio for the patient [43].

*Enterococcus* spp. resistance to ampicillin increased significantly from 7.2% to 16.3% in the study period (*p* = 0.013). The resistance rate to nitrofurantoin remained below 5–10% and only several cases of vancomycin-resistant enterococcus (VRE) were detected, mostly in recent years (*p* for trend 0.018). This compares favourably with a British study, where 40% of *Enterococcus spp.* isolates showed resistance to nitrofurantoin and a prevalence of VRE was 9.8% [44]. Magyar et al. [33] reported a much lower resistance rate to ampicillin, which may be explained by the inclusion of both *E. faecalis* and *E. faecium* in our study, the latter being often ampicillin resistant.

The main limitation of the present study is the impossibility to differentiate community- and hospital-acquired infections as the dates of urine cultures could not be linked to admission and discharge dates of each inpatient episode. It might also be insightful to discriminate urine samples representing asymptomatic bacteriuria as opposed to a clinical UTI, but the nature of our retrospective data would not allow for this. Unfortunately, this trait is common to most other AMR reports in the literature.

The present survey fulfils the recommendation of the European Association of Urology guidelines [45] to monitor local resistance patterns of common uropathogens so that empirical antibiotic treatment can be tailored to the local epidemiological situation. Ideally, these collected data should be gathered in a broader survey, such as the global prevalence of infections in urology (GPIU) [46] study, that allows for comparisons between countries and institutions and plays an important role in the monitoring of global resistance trends.

Our data suggest only a handful of options exist for the treatment of patients with a complicated UTI. In everyday practice, however, we do keep in mind the threat of AMR and avoid the empirical use of carbapenems and piperacilin/tazobactam despite their favourable resistance profiles seen on our data. Empirical treatment of newly admitted UTI patients starts with a third-generation cephalosporine +/− gentamicin. Higher-class antimicrobials are reserved for culture-proven non-susceptibility to standard antibiotic regimens and only rarely used without a culture in clinically deteriorating patients.

## 4. Materials and Methods

The Department of Clinical Microbiology electronic database was searched and all urinary cultures between January 2011 and December 2019 were reviewed. These included midstream urine cultures, urine sampled during theatre procedures, from indwelling catheters, suprapubic catheters, nephrostomy tubes, and urine samples from uretero-ileostomies. Only inpatient samples were considered for analysis. Duplicates were excluded, allowing only one isolate of a given pathogen per patient per year. Prevalence of major uropathogenic organisms and their antimicrobial susceptibility patterns were analysed.

Antimicrobial resistance patterns for five most common uropathogens are reported here: *Escherichia coli* (*E. coli*), including subspecies *E. coli haemolytica*, *Enterococcus* spp. (including both *E. faecalis* and *E. faecium*), *Klebsiella* spp., *Pseudomonas aeruginosa* (*P. aeruginosa*), and *Proteus spp.* (including *P. mirabilis, P. vulgaris*, and other species).

### Culture Methods and Susceptibility Testing

Urine samples were processed by a semi-quantitative dilution method. Uncentrifuged urine was inoculated with a 0.01-mL loop on blood and UriSelect chromogenic agar (Bio-Rad, Berkeley, CA, USA). Diluted urine 1:10 in saline was inoculated with a 0.01-mL and 0.001-mL loop on Columbia blood agar (Bio-Rad, Berkeley, CA, USA) and UriSelect chromogenic agar. Agar plates were incubated at 37 °C for 20–24 h. Detected pathogens in significant amounts were identified according to phenotypical characteristics (growth properties, biochemical tests) and using the semi-automatic system MIKROLATEST ID (Erba-Lachema, Brno, Czech Republic). Antibiotic susceptibility testing was performed by the disc diffusion method on Mueller-Hinton agar plates (Bio-Rad, Berkeley, CA, USA) and the MIC dilution method (TRIOS MIC, Prague, Czech Republic until 2017, then MICRO-LA-TEST ATB (MIC) Erba-Lachema, Brno, Czech Republic). Results were evaluated according to EUCAST MIC breakpoint tables. Intermediate results (newly termed “susceptible, increased exposure”) were excluded from analysis.

The following antimicrobial agents were selected for the survey: ampicillin, amoxicillin/clavulanate and piperacillin/tazobactam to include an unprotected penicillin, a penicillin-beta-lactamase inhibitor combination and an antipseudomonad penicillin with a beta-lactamase inhibitor; cefuroxime, cefotaxime (2nd and 3rd generation cephalosporine, respectively), ceftazidime and cefepime (3rd generation with antipseudomonad coverage and 4th generation, respectively); ciprofloxacin and ofloxacin to represent fluoroquinolones, gentamicin and amikacin for aminoglycosides; the carbamenems ertapenem, meropenem, and imipenem; and trimethoprim-sulfamethoxazole (co-trimoxazole), nitrofurantoin, colistin, and vancomycin.

The Cochrane-Armitage test was used to assess the statistical significance of trends. Statistical analyses were performed in XLSTAT 2020.1.3 (Addinsoft, New York, NY, USA). Alpha level of 0.05 was considered significant. Ethical approval was waived for this retrospective analysis of anonymised data.

## Figures and Tables

**Table 1:** Cumulative resistance rates for individual uropathogens and antibiotics, respectively, 2011–2019. N, total number of isolates; n, number of resistant isolates; NT, not tested. Percentages represent the rate of resistant isolates.

Uropathogen	Antimicrobial Agent							
	**Ampicillin**		**Amoxicillin/clavulanic acid**		**Piperacillin/tazobactam**			
	*n/N*		*n/N*		*n/N*			
*E. coli*	641/1149	55.8%	163/1138	14.3%	50/358	14.0%		
*Klebsiella spp.*	522/522	100.0%	191/492	38.8%	129/245	52.7%		
*P. aeruginosa*	NT		NT		55/180	30.6%		
*Proteus spp.*	138/241	57.3%	30/239	12.6%	1/113	0.9%		
*Enterococcus spp.*	80/756	10.6%	NT		NT			
	**Cefuroxime**		**Cefotaxime**		**Ceftazidime**		**Cefepime**	
	*n/N*		*n/N*		*n/N*		*n/N*	
*E. coli*	143/1143	12.5%	108/1135	9.5%	100/1131	8.8%	86/367	23.4%
*Klebsiella spp.*	219/523	41.9%	173/505	34.3%	180/503	35.8%	151/260	58.1%
*P. aeruginosa*	NT		NT		47/252	18.7%	56/178	31.5%
*Proteus spp.*	38/242	15.7%	14/240	5.8%	7/240	2.9%	8/109	7.3%
*Enterococcus spp.*	NT		NT		NT		NT	
	**Ciprofloxacin**		**Ofloxin**					
	*n/N*		*n/N*					
*E. coli*	161/382	42.1%	267/1015	26.3%				
*Klebsiella spp.*	191/278	68.7%	183/460	39.8%				
*P. aeruginosa*	99/260	38.1%	12/12	100.0%				
*Proteus spp.*	55/113	48.7%	107/210	51.0%				
*Enterococcus spp.*	NT		NT					
	**Gentamicin**		**Amikacin**					
	*n/N*		*n/N*					
*E. coli*	82/1149	7.1%	5/381	1.3%				
*Klebsiella spp.*	170/521	32.6%	7/278	2.5%				
*P. aeruginosa*	81/258	31.4%	24/258	9.3%				
*Proteus spp.*	70/242	28.9%	0/112	0.0%				
*Enterococcus spp.*	NT		NT					
	**Ertapenem**		**Meropenem**		**Imipenem**			
	*n/N*		*n/N*		*n/N*			
*E. coli*	1/377	0.3%	0/388	0.0%	0/385	0.0%		
*Klebsiella spp.*	9/276	3.3%	0/283	0.0%	0/282	0.0%		
*P. aeruginosa*	NT		54/170	31.8%	21/135	15.6%		
*Proteus spp.*	0/112	0.0%	0/114	0.0%	1/114	0.9%		
*Enterococcus spp.*	NT		NT		NT			
	**Vancomycin**		**Colistin**		**Nitrofurantoin**		**Cotrimoxazole**	
	*n/N*		*n/N*		*n/N*		*n/N*	
*E. coli*	NT		1/123	0.8%	47/973	4.8%	398/1150	34.6%
*Klebsiella spp.*	NT		2/100	2.0%	144/313	46.0%	248/522	47.5%
*P. aeruginosa*	NT		0/260	0.0%	NT		NT	
*Proteus spp.*	NT		NT		173/173	100.0%	162/242	66.9%
*Enterococcus spp.*	8/796	1.0%	NT		34/711	4.8%	NT	

## Supplementary Materials

The following are available online at https://www.mdpi.com/2079-6382/9/9/630/s1. Table S1. Resistance rates against penicillin derivatives. The table shows resistance rates against ampicillin, amoxicillin/clavulanic acid and piperacillin/tazobactam 2011–2019. *Klebsiella spp*. is inherently resistant to ampicillin. N, total number of isolates; n, number of resistant isolates; NS, trend not significant. Percentages represent the rate of resistant isolates * Note small number of isolates in some or all years when interpreting statistical significance. ^†)^ Statistical significance of trend is retained when only 4 consecutive years with *N* > 30 are included in analysis. Table S2. Resistance rates against cephalosporines. The table shows resistance rates against cefuroxime, cefotaxime, ceftazidime and cefepime 2011–2019. * Note small number of isolates in some or all years when interpreting statistical significance. ^‡)^ Statistical significance of trend is lost when only 4 consecutive years with *N* > 30 are included in analysis. Table S3. Resistance rates against fluoroquinolones. The table shows resistance rates against ciprofloxacin and ofloxacin 2011–2019. * Note small number of isolates in some or all years when interpreting statistical significance. ^†)^ Statistical significance of trend is retained when only 4 consecutive years with *N* > 30 are included in analysis. Table S4. Resistance rates against aminoglycosides. The table shows resistance rates against gentamicin and amikacin 2011–2019. * Note small number of isolates in some or all years when interpreting statistical significance. Table S5. Resistance rates against carbapenems. The table shows resistance rates against ertapenem, meropenem and imipenem 2011–2019. * Note small number of isolates in some or all years when interpreting statistical significance. Table S6. Resistance rates against cotrimoxazol and nitrofurantoin. The table shows resistance rates against cotrimoxazol and nitrofurantoin 2011–2019. *Proteus spp*. is inherently resistant to nitrofurantoin. * Note small number of isolates in some or all years when interpreting statistical significance. ^‡)^ Statistical significance of trend is lost when only 5 consecutive years with *N* > 30 are included in analysis. Table S7. Resistance rates against vancomycin and colistin. The table shows resistance rates against vancomycin and colistin 2011–2019. Figure S1a–e: Statistically significant trends in antimicrobial resistance 2011–2019 for *E. coli, Klebsiella spp., Proteus spp., P. aeruginosa* and *Enterococcus spp*., respectively. The figures should be interpreted along with the Supplementary Material Tables S1–S7. AMP ampicillin, AMC amoxicillin/clavulanic acid, PPT piperacillin/tazobactam, CRX cefuroxime, CPM cefepime, CIP ciprofloxacin, OFL ofloxacin, GEN gentamicin, AMI amikacin, MER meropenem, NTF nitrofurantoin, VAN vancomycin.
