# Supplementary figures and images for: Current Antibiotic Resistance Trends of Uropathogens in Central Europe: Survey from a Tertiary Hospital Urology Department 2011–2019
Source: Antibiotics (Basel). 2020 Sep 22;9(9):630. doi: 10.3390/antibiotics9090630 (PMC7559630; doi:10.3390/antibiotics9090630)

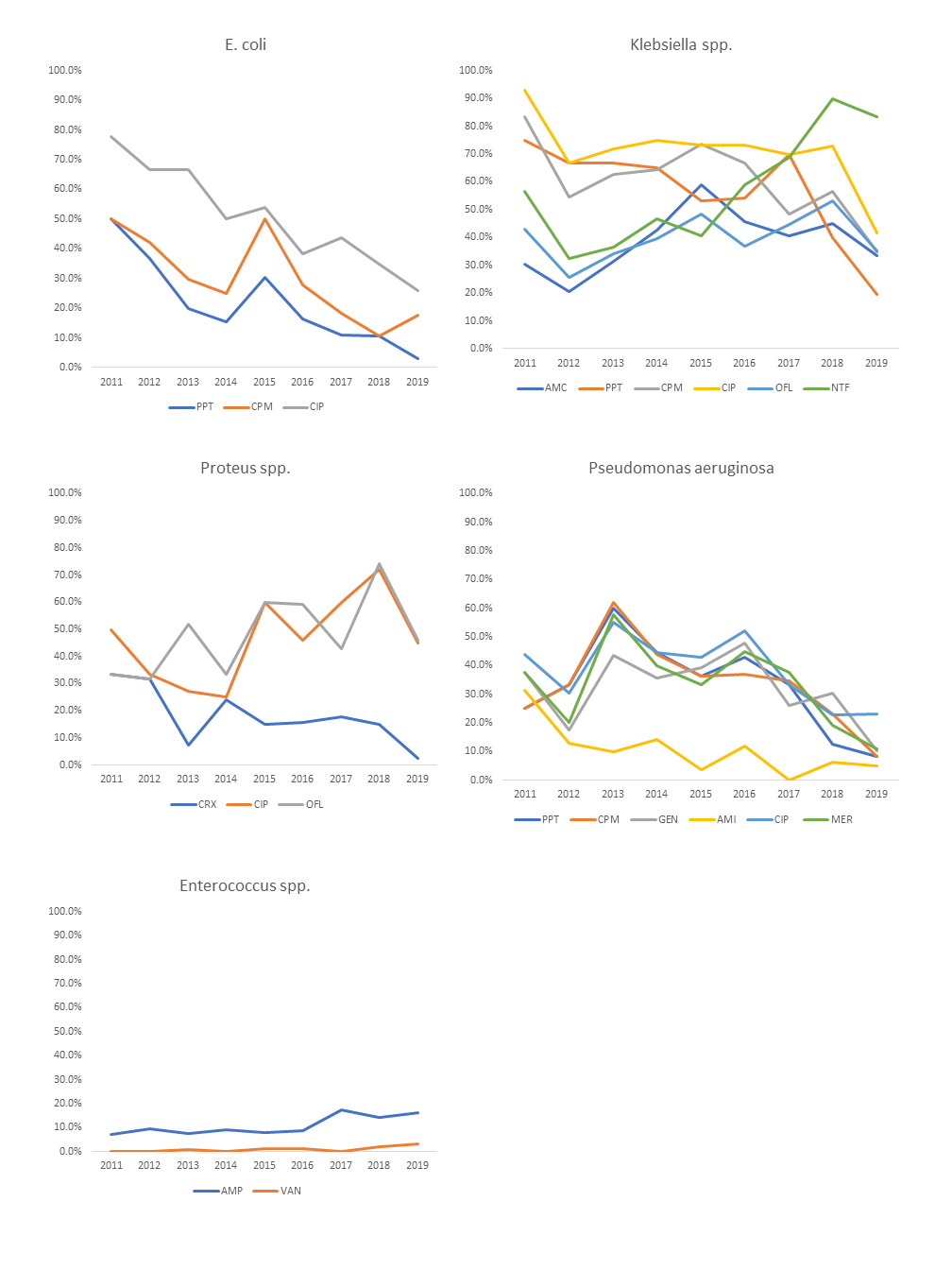

Supplement: Supplementary file 1 [file antibiotics-09-00630-s001.zip › Hrbacek_Figure_S1a-e.png]
